# Mapping the Presence of Anxiety Symptoms in Adults With Major Depressive Disorder

**DOI:** 10.3389/fpsyt.2021.595418

**Published:** 2021-05-19

**Authors:** Fenfen Ge, Jingwen Jiang, Yue Wang, Mentong Wan, Wei Zhang

**Affiliations:** ^1^Mental Health Center, West China Hospital, Sichuan University, Chengdu, China; ^2^West China Biomedical Big Data Center, West China Hospital, Sichuan University, Chengdu, China; ^3^Wuyuzhang Honors College, Sichuan University, Chengdu, China

**Keywords:** major depressive disorder, anxiety, co-occurrence, network analysis, psychopathology

## Abstract

**Background:** Patients with major depressive disorder (MDD) often present with co-occurring anxiety symptoms. The network method provides a novel view on understanding the co-occurrence of depressive and anxiety symptoms. Thus, the purpose of our study was to explore it by applying network analysis methods.

**Methods:** We used electronic medical records from West China Hospital in China. In total, 3,424 patients who met the criteria for MDD were included. R-studio 3.6 was used to estimate the network structure. First, we estimated the network structure of depression and anxiety symptoms using the graphic LASSO algorithm. Then, we estimated the centrality indices of nodes to determine which symptoms are more central in the network. We then estimated the bridge centrality indices using the *bridge* function via the R package *networktools*.

**Results:** Some strong connections were found like “easy to wake up,” “wake up early,” and “difficulty falling asleep,” “suicidal thoughts,” and “hopelessness.” “Depressed mood,” “somatic anxiety,” “hopelessness,” “anxiety mood,” and “tension” have the higher centrality indices. Results revealed eight bridge symptoms (e.g., concentration/memory difficulty, gastrointestinal symptoms) in the co-occurrence network structure.

**Conclusions:** This research suggests that the described approach in mapping the presence of anxiety symptoms in individuals with major depression might potentially increase diagnostic precision and help choose more targeted interventions and potentially reduce the occurrence of treatment resistance.

## Introduction

*Major depressive disorder* (MDD) is a debilitating psychiatric condition characterized by depressed mood, decreased energy level, and lack of interest in pleasurable activities (1). It has a significant burden on both individuals and society and is related to psychological impairments and health dysfunction. Among Chinese adults, MDD has a 12-month and lifetime prevalence rate of 3.6 and 6.9%, respectively (2). Gaspersz's study showed that 40–60% of patients with MDD also have anxiety symptoms (3). Another survey suggested that 50% of individuals with MDD meet the diagnostic criteria for anxiety disorders (4, 5).

The co-occurrence anxiety symptoms among patients with MDD have important clinical implications. Firstly, the co-occurrence anxiety symptoms predict a more chronic course and more severe disease progression (6). Secondly, compared with individuals who have MDD without anxiety symptoms, patients influenced by anxiety and depressive symptoms have shown greater functional disability, poorer quality of life, and higher risk of suicide behavior (7, 8). Thirdly, MDD with prominent coexisting anxiety symptoms is more difficult to treat than MDD without anxiety symptoms (9). Fourth, anxiety symptoms are associated with occupation of more health care resources among patients with MDD (10). Previous studies either concentrated on the count symptoms to clarify a diagnosis and calculate the prevalence rate of comorbidity or regarded the symptoms as indicators of latent dimensions, however, the between-symptom links are considered a byproduct of dimensional community (11).

Network theory provides us a novel perspective of mental disorders. Network analysis conceptualizes symptoms as constituents of mental disorders, as compared to traditional methods that assume an underlying disease in advance as the common cause of symptoms. Recently, network analysis models are rapidly growing, not only concerning methodological issues but also in offering an appealing interpretation of psychopathology (12, 13).

From the perceptive of topology, network structure consists of nodes(symptoms) and edges(association between symptoms) (14). The importance of nodes was evaluated via centrality indices. Edges represent the links between pairs of symptoms and thicker edges denote larger correlations (15). Specifically, centrality indices include strength, closeness, and betweenness that allow clinicians to discern the symptoms with the greatest importance in the network structure. Those with high centrality indices convey more clinical information (16, 17). Strength is one of the most commonly used centrality indices as it is easy to interpret and is the most stable centrality index (18). In the opinion of network theory, comorbidity is regarded as a constellation of symptom-level relationships (19). Symptoms that link two mental disorders are regarded as “bridge symptom.” Bridge symptoms indicated by bridge centrality indices mainly included bridge strength, bridge closeness, and bridge betweenness (20). Bridge symptoms may have an important role in the development and maintenance of co-occurring mental disorder (21). Thus, when one mental disorder presents, intervention on potential bridge symptoms may contribute to preventing co-occurrence (20). For example, if we suppose that sleep disturbance is a bridge symptom between depression and anxiety, then patients who suffer poor sleep quality as one of their MDD symptoms would be at greater risk for anxiety compared to those without sleep disturbance. Thus, it would be wise for psychiatrists to treat these bridge symptoms therapeutically to reduce the co-occurrence.

At present, 10 studies utilized network analysis to explore comorbidity and co-occurrence in depression (22–31). Previous studies have explored the comorbidity between anxiety and depression (23), posttraumatic stress disorder and co-morbidity depressive symptoms (31), and comorbidity between obsessive-compulsive disorder and depression (26). While previous network researches in MDD have broadened our knowledge of the field, several limitations remain. Firstly, most studies use self-reported questionnaires (e.g., Patient Health Questionnaire-9 and General Anxiety Disorder-7) to assess symptoms among the general population (29, 30), while self-report questionnaires have a risk for response biases (32). Few studies used a sample that comprised treatment-seeking patients with MDD (23). Secondly, while sample size plays an important role in establishing a reliable network structure, most studies used a small sample size that ranges from 296 (25) to 1,029 (23, 33). Thirdly, no studies to date have explored the co-occurrence between MDD and anxiety symptoms based on real-world evidence. Real-word evidence is important for establishing the network structure of co-occurrence between MDD and anxiety symptoms. In this regard, electronic medical records (EMRs) bring new chances in clinical research, providing the potential chance for low-cost and high-volume data on clinical research (34).

The main aim of this study, which was based on the retrospective of EMRs, was to establish the co-occurring network structure between MDD and anxiety symptoms to identify the bridge symptoms.

## Methods

### Study Population

**Figure 1:**
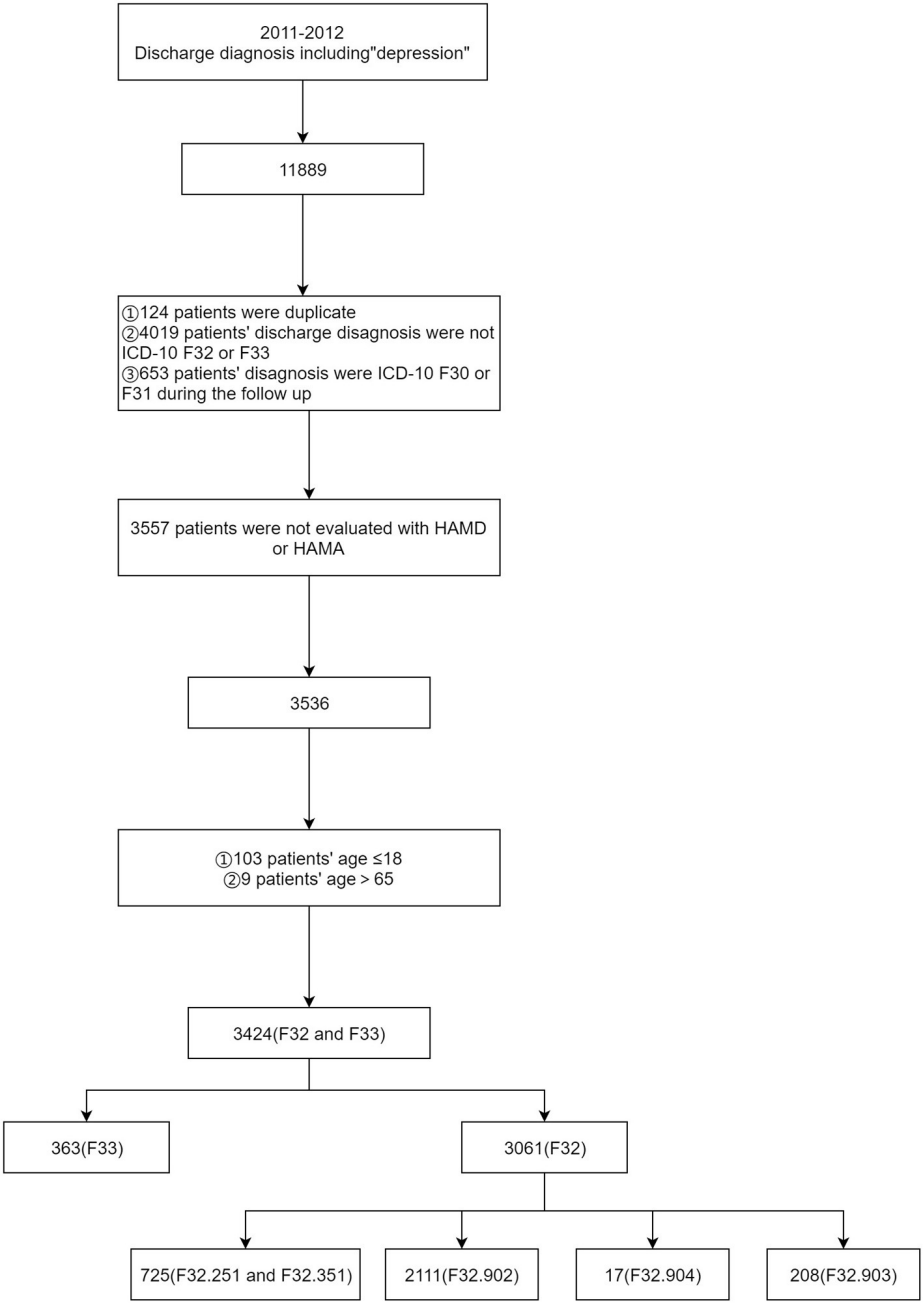
The process of data extraction.

This was a retrospective study based on pre-existing data derived from EMRs undertaken at West China Hospital. Eligible patients were those with a diagnosis of MDD, aged between 18 and 65. In this study, we identified MDD patients through recorded primary diagnosis at discharge and based on the International Classification of Disease, Tenth Revision (Clinical Modification Codes F32, single episode major depressive disorder and F33, recurrent major depressive disorder), which has been described in another study (35). We extracted anonymous clinical-related information. Exclusion criteria are the following 3 items. Patients' follow-up discharge diagnosis code is F30 or F31. Patients with MDD did not accomplish Hamilton Depression Scale-24 (HAMD-24) and Hamilton Anxiety Scale-14 (HAMA-14) at admission. ③ Age ≤ 18 or age > 65. The details are shown in Figure 1. The data used to support the findings of this manuscript are restricted by the West China Hospital in order to protect patient privacy and avoid legal and ethical risks. Data are available from the West China Hospital for researchers who meet the criteria for access to confidential data (data.cd120.com).

We obtained approval from the Ethics Committee of the West China Hospital, Sichuan University (2017 N0.185). As this is a retrospective study, Institute Review Board (IRB) waived the requirement for obtaining informed consent from the individual patients. We did not use information on the identity of patients and all related information was kept confidential. All procedures were in accordance with the ethical standards of the Ethics Committee and the revised Helsinki Declaration of 2008 (36, 37).

### Measures

HAMD-24 is one of the most widely used scales in clinical practical assessment of depression. It is assessed by psychiatrists and takes about 15 to 20 min to finish (38). HAMA-14 is one of the most widely used scales in the clinical practical assessment of anxiety. HAMA-14 includes 14 items with each item divided into 5 levels from 0 to 4 (39). The Chinese versions of HAMD-24 and HAMA-14 have good reliability and validity (40).

### Data Analysis

All analysis was accomplished using the R-3.6 studio. In this research, the missing data belong to the category of missing at random (MAR). So, we used the method of unconditional mean imputation to handle the missing data (41). The network structure consists of two elements: nodes and edges. Every node represents a symptom, and each edge demonstrates a relationship between the two symptoms. In our study, the nodes (symptoms) were represented the scale items of HAMD-24 and HAMA-14. We used a graphical Gaussian model to estimate the networks. In addition, we used the least absolute shrinkage and selection operator (LASSO) to regularize our model and used the *qgraph* package to visualize the network. Next, we computed the centrality indices (i.e., betweenness, strength, and closeness) of nodes to find which symptoms are more central in the network structure. Betweenness and closeness are often not reliably estimated (17). Thus, we only reported the strengths in this article, while other node centrality indices are provided in the Supplementary Material. We assessed the accuracy stability of the centrality using the bootstrap approach in the *bootnet* package. To gain a stable and interpretable centrality, the CS coefficient should be >0.25 (42). We estimated the stability of edge-weights by bootstrapping the 95% confidence intervals (CIs), where fewer overlaps in the CIs indicate higher stability. Jones uses the term community to demonstrate a theoretically based group of nodes that correspond to a mental disorder according to clinical criteria, instead of according to any network analytic procedure (20). This method contributes to identifying bridge nodes, especially when networks are large, complex, or difficult to account for visually. The *networktools* package (43) were used to calculate the values and create plots. You can get the code from the Git hub (https://github.com/fenfenge/Network-structure).

## Results

### Descriptive Statistics

We included 3,424 patients with MDD comprising 2,349 females and 1,075 males, with ages ranging from 18 to 65 (*M* = 42.5, SD = 13.25). Table 1 shows the items, item content, sample means, standard deviation, and missing items/percentage.

**Table 1:** Items, item content, missing items, means, and standard deviations for HAMD-24 and HAMD-14.

**Item**	**Item content**	**Missing items/Percentage**	**M**	**SD**
D1	Depressed or sad mood	0/0.00%	2.55	0.95
D2	Guilty feelings	0/0.00%	0.88	0.95
D3	Suicidal thoughts	0/0.00%	1.07	1.15
D4	Difficulty falling asleep	0/0.00%	1.55	0.97
D5	Wake up early	0/0.00%	1.37	0.91
D6	Early awakening	0/0.00%	1.28	0.95
D7	Loss of interest/pleasure	0/0.00%	1.98	1.02
D8	Psychomotor retardation	0/0.00%	0.95	0.93
D9	Agitation	0/0.00%	0.61	0.88
D10	Nervousness or anxiety	0/0.00%	1.80	1.03
D11	Somatic anxiety	0/0.00%	1.55	1.07
D12	Gastrointestinal symptoms	2/0.06%	0.78	0.88
D13	Somatic symptoms	2/0.06%	0.93	0.89
D14	Loss of interest in sex	5/0.15%	0.32	0.69
D15	Hypochondria	2/0.06%	0.63	0.90
D16	Loss of weight	2/0.06%	0.61	0.82
D17	Insight	2/0.06%	0.46	0.68
D18	Changes in mood patterns	2/0.06%	0.45	0.68
D19	Depersonalization/Derealization	7/0.20%	0.13	0.43
D20	Paranoid symptoms	8/0.23%	0.28	0.65
D21	Obsessive-compulsive symptoms	8/0.23%	2.00	0.55
D22	Feel less capable	8/0.23%	1.48	1.02
D23	Hopelessness	8/0.23%	1.38	1.07
D24	Inferiority hopelessness	8/0.23%	1.14	1.00
A1	Anxiety mood	52/1.52%	2.01	0.97
A2	Tension	51/1.49%	1.51	0.99
A3	Afraid	51/1.49%	1.22	1.01
A4	Insomnia	51/1.49%	1.85	1.30
A5	Concentration/memory difficulty	52/1.52%	1.18	0.92
A6	Pessimism	56/1.64%	2.26	0.97
A7	Muscular symptoms	60/1.75%	0.71	0.94
A8	Sensory symptoms	60/1.75%	0.87	0.98
A9	Cardiovascular symptoms	60/1.75%	0.87	0.97
A10	Respiratory symptoms	60/1.75%	0.57	0.82
A11	Gastrointestinal symptoms	61/1.78%	0.84	1.00
A12	Genitourinary symptoms	60/1.75%	0.36	0.69
A13	Automatic symptoms	60/1.75%	1.05	0.98
A14	Behavior during the talk	60/1.75%	1.23	0.99

### Network Estimation

**Figure 2:**
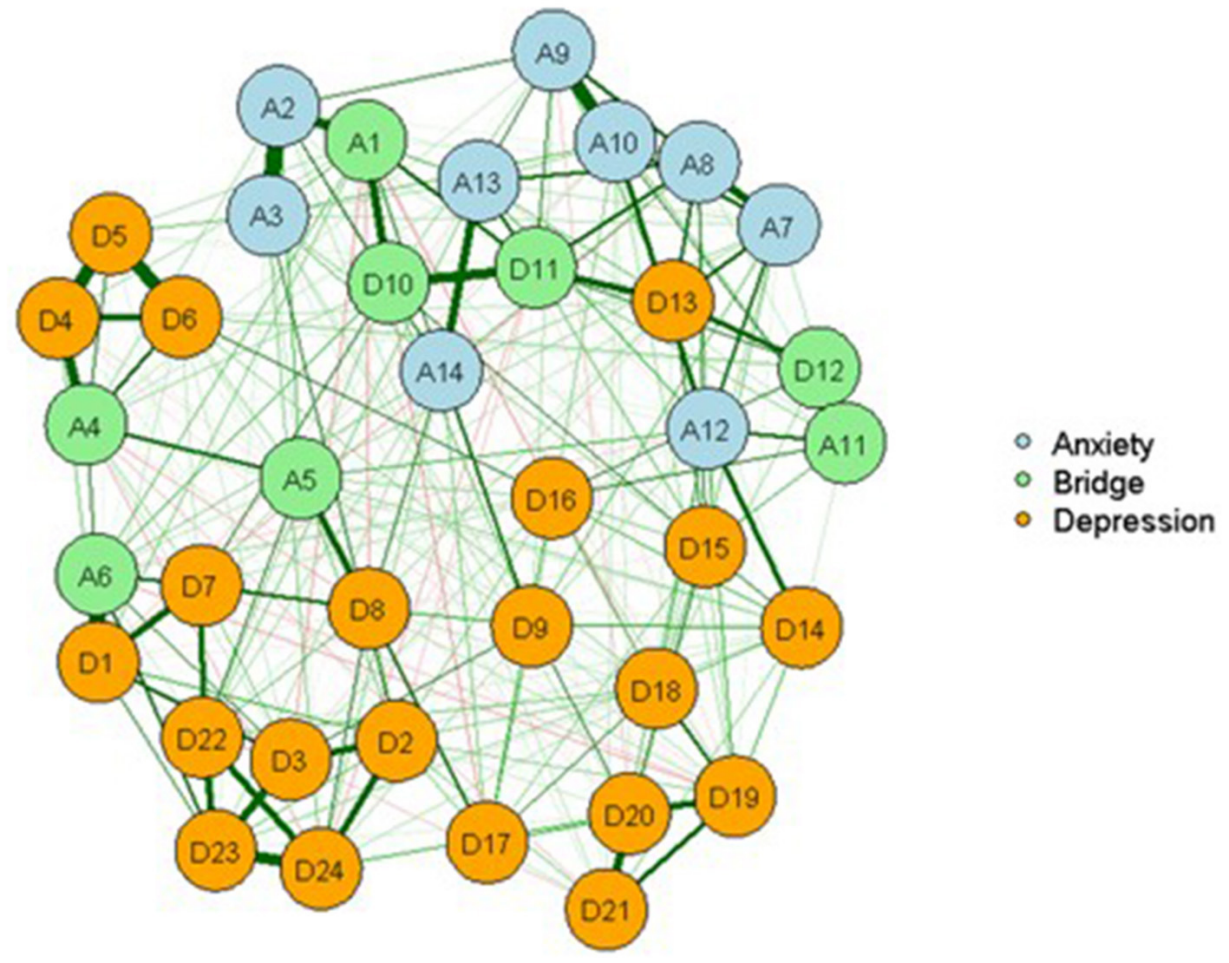
Network of MDD and anxiety symptoms showing bridge symptoms among clinical samples. D1: Depressed or sad mood; D2: Guilty feelings; D3: Suicidal thoughts; D4: Difficulty falling asleep; D5: Easy to wake up; D6: Early awakening; D7: Loss of interest/pleasure; D8: Psychomotor retardation; D9: Agitation; D10: Nervousness or anxiety; D11: Somatic anxiety; D12: Gastrointestinal symptoms; D13: Somatic symptoms; D14: Loss of interest in sex; D15: Hypochondria; D16: Loss of weight; D17: Insight; D18: Changes in mood patterns; D19: Depersonalization/Derealization; D20: Paranoid symptom; D21: Obsessive-compulsive symptom; D22: Feel less capable; D23: Hopelessness; D24: Inferiority hopelessness; A1: Anxiety mood; A2: Tension; A3: Afraid; A4: Insomnia; A5: Concentration/memory difficulty; A6: Pessimism; A7: Muscular symptoms; A8: Sensory symptoms; A9: Cardiovascular symptoms; A10: Respiratory symptoms; A11: Gastrointestinal symptoms; A12: Genitourinary symptoms; A13: Automatic symptoms; A14: Behavior during the talk.

The graphical LASSO network is shown in Figure 2. A thicker edge indicates a stronger association between the symptoms. Green edges represent positive regularized partial correlations, and red edges represent negative regularized partial correlations. Network analysis demonstrated that five strong connections edges were among the HAMD-24 items. The top edge was between the items “easy to wake up,” “wake up early,” and “difficulty falling asleep” (D4: D5: D6). Additionally, HAMD-24 items “suicidal thoughts” and “hopelessness” (D3:D23) and “hopelessness” and “inferiority” (D23:D24) were among the strongest. There were two strongest edges between the HAMA-14 items. The strongest edge was between items “tension” and “afraid” (A2:A3), followed by “cardiovascular symptoms” and “respiratory symptoms” (A9:A10).

### Network Inference and Stability

**Figure 3:**
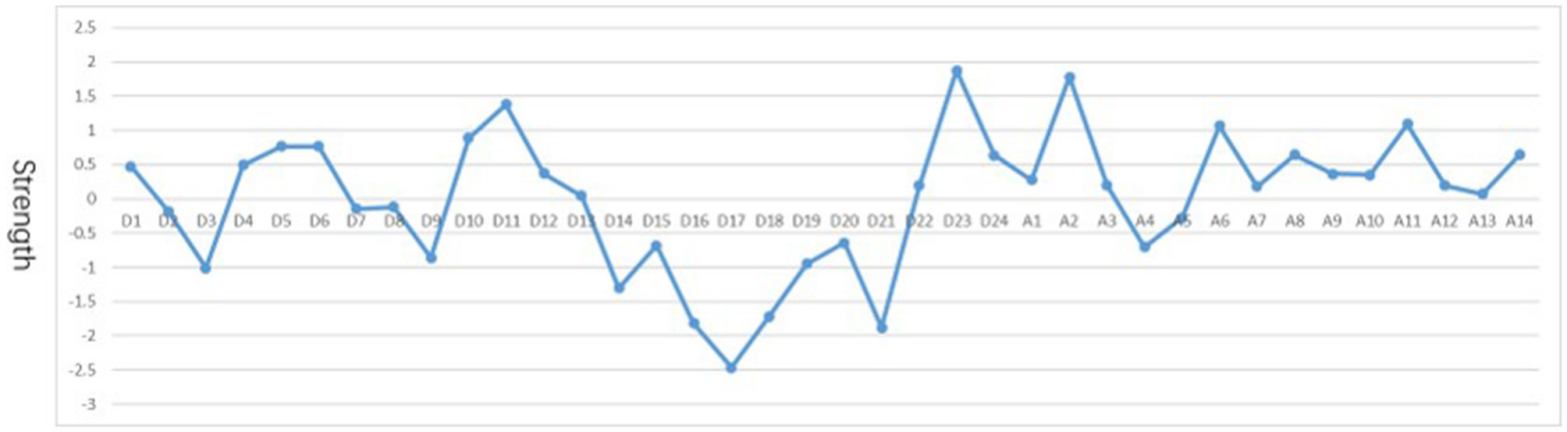
Plot of standardized centrality indices for network. D1: Depressed or sad mood; D2: Guilty feelings; D3: Suicidal thoughts; D4: Difficulty falling asleep; D5: Easy to wake up; D6: Early awakening; D7: Loss of interest/pleasure; D8: Psychomotor retardation; D9: Agitation; D10: Nervousness or anxiety; D11: Somatic anxiety; D12: Gastrointestinal symptoms; D13: Somatic symptoms; D14: Loss of interest in sex; D15: Hypochondria; D16: Loss of weight; D17: Insight; D18: Changes in mood patterns; D19: Depersonalization/Derealization; D20: Paranoid symptom; D21: Obsessive-compulsive symptom; D22: Feel less capable; D23: Hopelessness; D24: Inferiority hopelessness; A1: Anxiety mood; A2: Tension; A3: Afraid; A4: Insomnia; A5: Concentration/memory difficulty; A6: Pessimism; A7: Muscular symptoms; A8: Sensory symptoms; A9: Cardiovascular symptoms; A10: Respiratory symptoms; A11: Gastrointestinal symptoms; A12: Genitourinary symptoms; A13: Automatic symptoms; A14: Behavior during the talk.

Strength centrality is shown in Figure 3. Firstly, nodes demonstrating “depressed or sad mood” (D1), “somatic anxiety” (D11), and “hopelessness” (D23) were among the MDD symptoms exhibiting higher levels of strength. Secondly, “anxiety mood” (A1) and “tension” (A2) were among the anxiety symptoms exhibiting higher levels of strength. In terms of stability of network analysis, bootstrap 95% CI demonstrated moderate stability for the strength index (Supplementary Materials).

**Figure 4:**
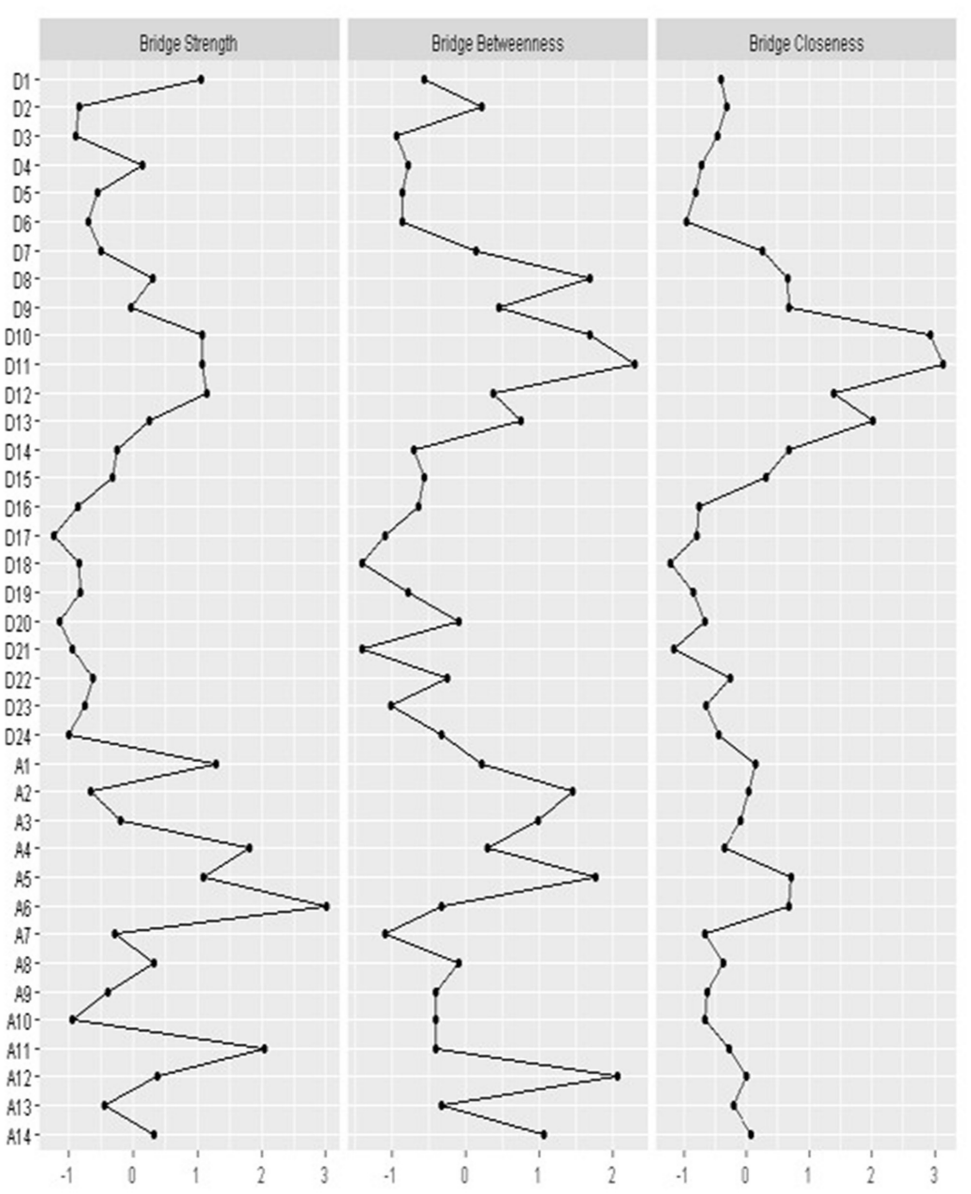
Bridge centrality indices of network structure of MDD and anxiety symptoms.

Bridge strength is shown in Figure 4. Nodes demonstrating “anxiety mood” (A1), “insomnia” (A4), “concentration/memory difficulty” (A5), “pessimism” (A6), and “gastrointestinal symptoms” (A11) were anxiety symptoms displaying higher levels of bridge strength on all MDD symptoms. The MDD symptoms “nervousness or anxiety” (D10), “somatic anxiety” (D11), and “gastrointestinal symptoms” (D12) exert a strong bridging effect on anxiety symptoms.

## Discussion

Many patients with MDD also experience anxiety symptoms. As far as we know, this is the first research to explore network connectivity among treat-seeking patients with MDD and co-occurrence anxiety symptoms based on real-world evidence. Perhaps the most prominent result was that not all nodes(symptoms) were equally important in the network with co-occurrence anxiety symptoms among patients with MDD (44, 45). Besides, we also found some strong linkage between symptoms, such as “easy to wake up” (D4), “wake up early” (D5), and “difficulty falling asleep” (D6), which were closely associated with each other.

Moreover, “depressed or sad mood” (D1) and “hopelessness” (D23) have the highest centrality in the network structure. Such results do not come as a surprise; previous studies that used self-report questionnaires and composite international diagnostic interviews also suggested that sad mood and hopelessness carry more weight than other symptoms of MDD (14, 46–48). “Somatic anxiety” (D11) had one of the highest centrality indices, making it a hallmark symptom of MDD among Chinese. “Somatic anxiety” is involved via overt signs of excessive autonomic activity and/or skeletal/muscle-motor tension. Due to the following three reasons that including (1) emotional symptoms are stigmatized, (2) thinking deviated far from internal experiences, (3)a special perspective of the self is emphasized, (49) somatization has been a general symptom in the Chinese population (50, 51). It is worthy that higher somatization was related to poorer performance in the cognitive and poorer antidepressant treatment clinical outcome (52). Anxiety symptoms (e.g., anxiety mood and tension) also had higher centrality. Goghari's 2-year longitudinal study found that patients with MDD reported higher levels of anxiety than those with other mental disorders. To some extent, easing the patient's anxiety level helps improve the outcome and daily function of depression (53).

Network theory helps to understand the co-occurrence of psychopathology. We could reduce co-occurrence by effectively “burning the bridge symptoms” between disorders (45). In this study, the MDD nodes that displayed the strongest association with anxiety symptoms were “nervousness” (D10), “somatic anxiety” (D11), and “gastrointestinal symptoms” (D12). Conversely, the anxiety nodes that displayed the strongest connection with MDD symptoms were “anxiety mood” (A1), “insomnia” (A4), “concentration/memory difficulty” (A5), “pessimism” (A6), and “gastrointestinal symptoms” (A11). Gastrointestinal symptoms were bridge symptoms that link MDD to anxiety symptoms and similarly linked anxiety symptoms to MDD. Gastrointestinal symptoms are common features for both MDD and anxiety disorders. They are also prominent features in posttraumatic stress disorder, schizophrenia, and autism spectrum disorder (54–56), demonstrating that gastrointestinal symptoms may be a common feature associated with a multitude of mental disorders. Simpson's review indicated that the role of gut microbiota in mood regulation and emotional processing may be of particular relevance to depression and anxiety etiology (57). In this study, concentration/memory difficulty was a bridge symptom that linked anxiety to MDD. Pettit's research found that computer-based attention training can reduce anxiety symptoms among the youth (58). These results suggested that attention training could be used to ease anxiety symptoms among MDD patients.

We found a strong connection between “easy to wake up,” “wake up early,” and “hard to fall asleep” (D4: D5: D6). Interestingly, the three symptoms above belong to the diagnostic criteria for insomnia (59) and MDD patients with sleep complaints are prone to more severe symptoms (60). Thus, insomnia is a valuable therapeutic target in MDD patients. The theoretical promise of network analysis is that psychiatric symptoms are not isolated and may accentuate each other (61). Symptoms may reinforce one another via creating a feedback loop, such as, “inferior helplessness” (D24) could lead to “hopelessness” (D23), and at the same time prompt “suicidal thoughts” (D3). These links may contribute to the build of a self-sustained symptom constellation. Thus, it is necessary to find potential self-sustaining mechanisms and timely interventions for feedback loops (13). Somatic symptoms (e.g., cardiovascular symptoms and respiratory symptoms) were closely connected. This can be explained by the mind-body interaction model, which states that information flows not only from the body to the brain but also from the brain to the body. In the brain, “prediction” is derived from metacognition (conceptual knowledge), namely higher-order thoughts and cognition, which helps estimate the generation of behavioral commands (62).

### Clinical Implication

The network model provides a novel view to investigate the potential mechanisms underlying the etiology and maintenance of mental disorders. We can understand how the symptoms are interrelated via networks and how to intervene on comorbidities. Firstly, when depressive patients are admitted to the hospital, psychiatrists should prioritize the evaluation of identified bridging MDD symptoms to screen patients with a higher risk of suffering anxiety symptoms. Secondly, from the viewpoint of network structure, we can intervene mental disorders from the following aspects: (1) symptoms (nodes) interventions: direct intervention of one or more symptoms; (2) network interventions: intervention symptom-symptom connections (61); (3) bridge symptoms: intervention of bridge symptoms to prevent co-occurrence. For example, we can intervene on closely connected sleep symptoms to avoid using certain antidepressants that may cause or even worsen sleep problems (63), since improving sleep contributes to improving the outcomes of MDD (64, 65). Our findings may be used to demonstrate which symptoms (e.g., gastrointestinal symptoms, insomnia) drive the association, and which should be handled first to reduce MDD and anxiety co-occurrence. These findings emphasize the importance of assessing anxiety symptoms among patients with MDD. Owing to the limitations of earlier versions of the Diagnostic and Statistical Manual of Mental Disorder (DSM) on grading targeting rules, anxiety in mental disorders has been underestimated, underdiagnosed, and undertreated. CBT is very effective for the treatment of anxiety symptoms and sleep disturbance (66). Thus, patients with MDD can be treated with suitable medicines and CBT.

### Strengths and Limitations

This research has several key strengths. First, we employed a sample comprised of treatment-seeking patients diagnosed with MDD according to the ICD-10. Moreover, HAMD and HAMA are evaluated by psychiatrists; thus, ambiguous/vague answers to the self-assessment scale were avoided. Second, we used a relatively large sample that contributed to establishing a reliable network structure with robust edge weights and centrality (42). To date, only one study has used large psychiatric samples of over 1,000 participants (23).

Despite the strengths, some limitations should be considered in this study. Firstly, this is a cross-sectional study, and we cannot explore changes in the co-occurrence network structure over time. Specifically, we are not sure whether the connections between symptoms appear temporarily or continuously, and what will happen to those strong connections under the intervention of strategy. Considering that network modes require the estimation of many parameters and these models need power to reliably detect small coefficients, exploration of larger data sets is necessary (67, 68). Thus, in this study, we do not compare the network structure in different severity of subgroup. Secondly, in our study, the target population was patients with MDD diagnosis so that the results could be generalized to the whole population. Specifically, the results do not suit individuals who suffer from certain depression symptoms, while failing to meet the diagnostic criteria for MDD. Third, Gureje's study showed that the presentation of mental disorders is influenced by culture and social milieu (69), while we only focused on Chinese patients. Future studies should consider the cross-cultural variation and explore how culture influences the presentation of mental disorders. Finally, we included patients with ICD-10 F32 and F33 diagnoses only. We did not check whether the patients had a comorbidity diagnosis with an anxiety disorder or any other co-morbidity. However, Wise's study (70) may support the notion that comorbidities might not be so important, at least concerning functional connectivity. Specifically, Wise's research supports the notion that biological abnormalities in functional connectivity in major depression across independent samples might overlap irrespective of the presence of anxiety comorbidities.

## Conclusion

This research is the first to explore the association between depressive and anxiety symptoms among MDD patients based on EMRs, thus offering an essential basis on how the two disorders co-vary. We found that some high central symptoms (e.g., hopelessness, somatic anxiety, and tension) and some bridge symptoms (e.g., concentration/memory difficulty, pessimism, and gastrointestinal symptoms). We summarize the evidence from the current research that treatment for co-occurring anxiety symptoms among MDD patients at symptom level may be efficacious. Multiple interventions, such as improving sleep, CBT, or attention training, could be applied to address these co-occurring symptoms.

## Data Availability Statement

The data analyzed in this study is subject to the following licenses/restrictions: The dataset belong to the West China Hospital, Sichuan University. Requests to access these datasets should be directed to Jingwen Jiang, jiangjingwen@wchscu.cn.

## Ethics Statement

The studies involving human participants were reviewed and approved by Ethics Committee of the West China Hospital, Sichuan University. The ethics committee waived the requirement of written informed consent for participation.

## Author Contributions

FG participated in the study design, data analysis, interpretation of findings, literature search, writing, implementation, and approval of the final manuscript. WZ conceived and designed the study. JJ, YW, and MW participated in the study data analysis. All authors have approved the final manuscript.

## Conflict of Interest

The authors declare that the research was conducted in the absence of any commercial or financial relationships that could be construed as a potential conflict of interest.
