# Supplementary figures and images for: Mapping the Presence of Anxiety Symptoms in Adults With Major Depressive Disorder
Source: Front Psychiatry. 2021 May 19;12:595418. doi: 10.3389/fpsyt.2021.595418 (PMC8169985; doi:10.3389/fpsyt.2021.595418)

● Bootstrap mean    ● Sample

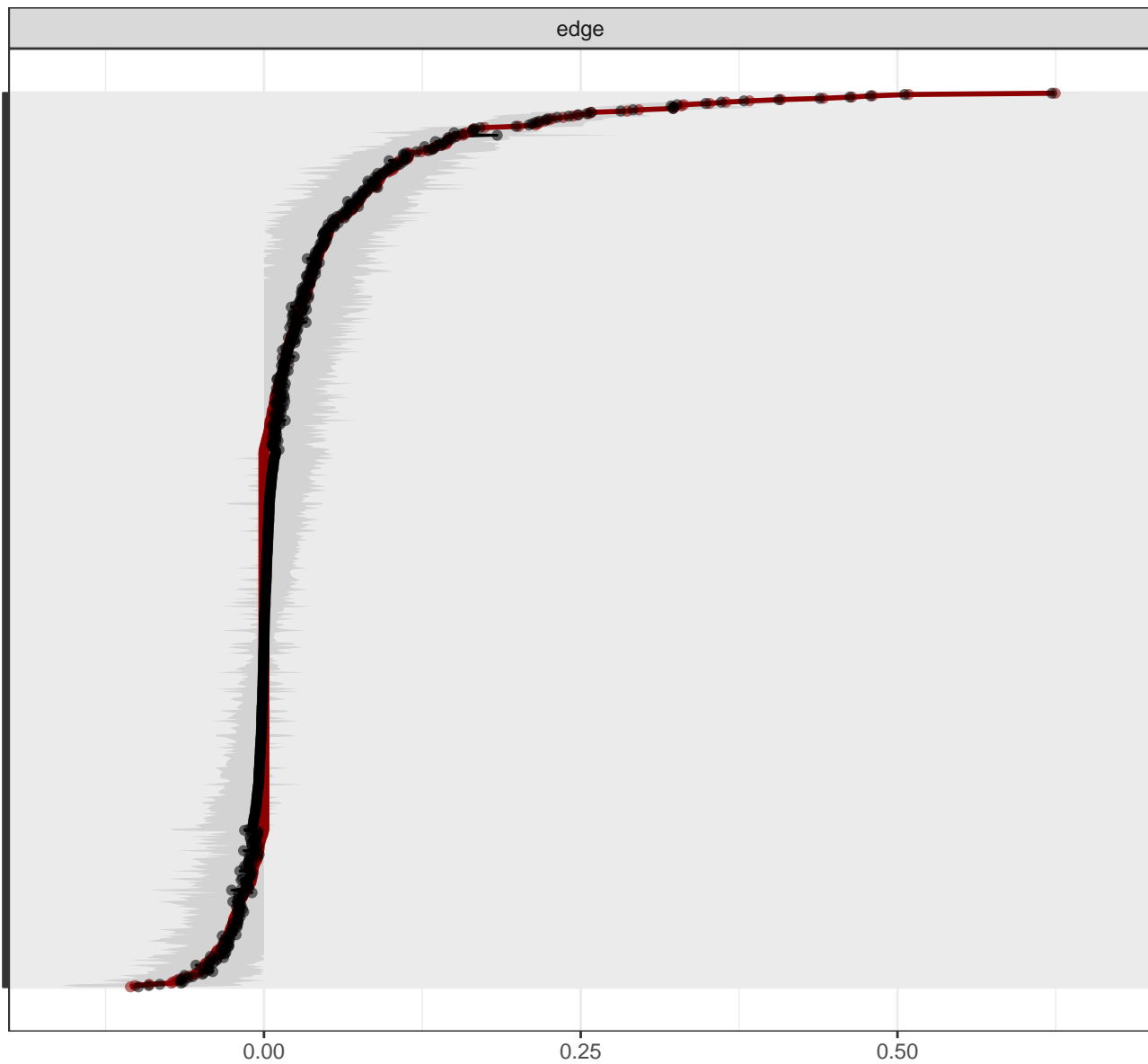

Supplement: Supplementary file 1 [file Data_Sheet_1.PDF]

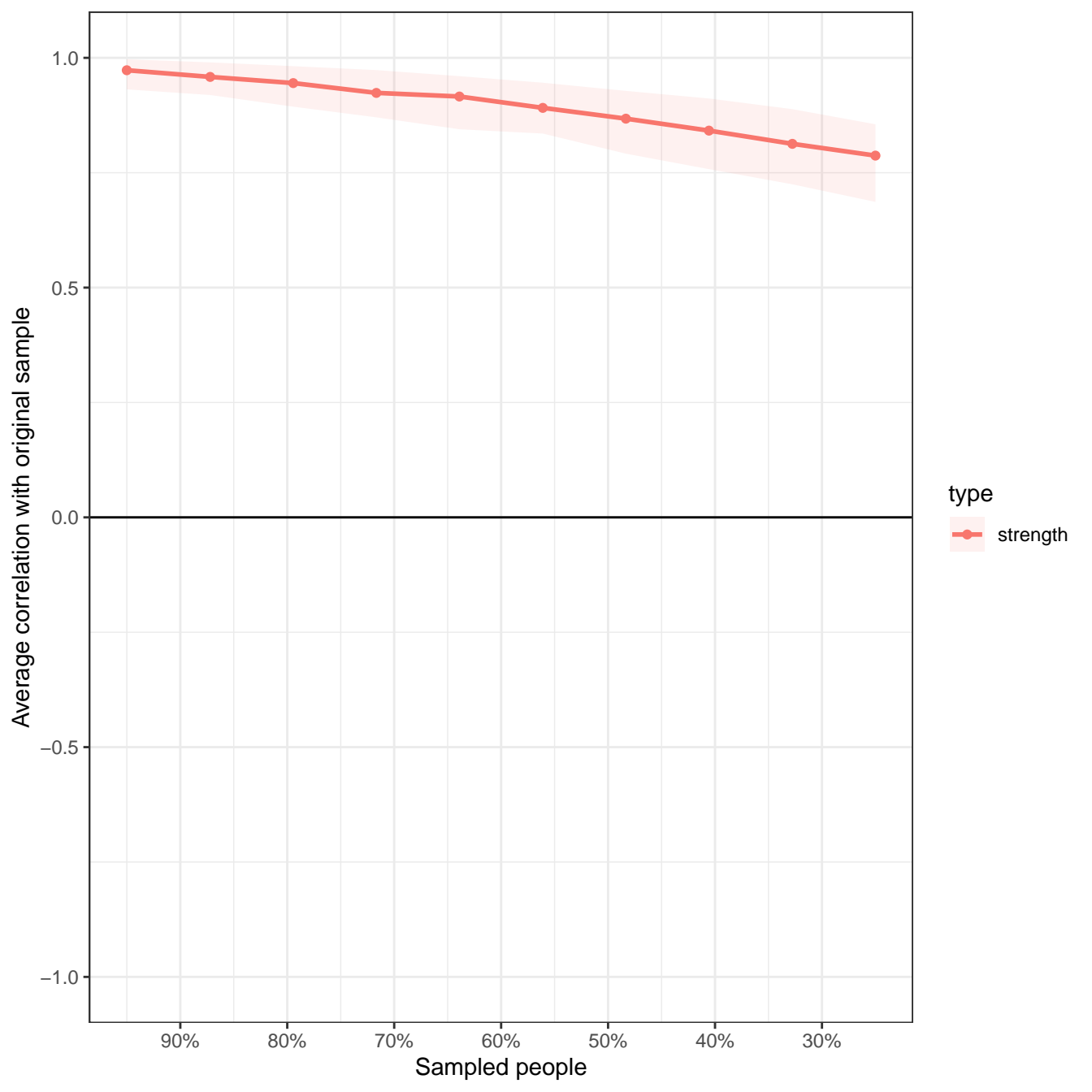

Supplement: Supplementary file 2 [file Data_Sheet_2.PDF]

edge

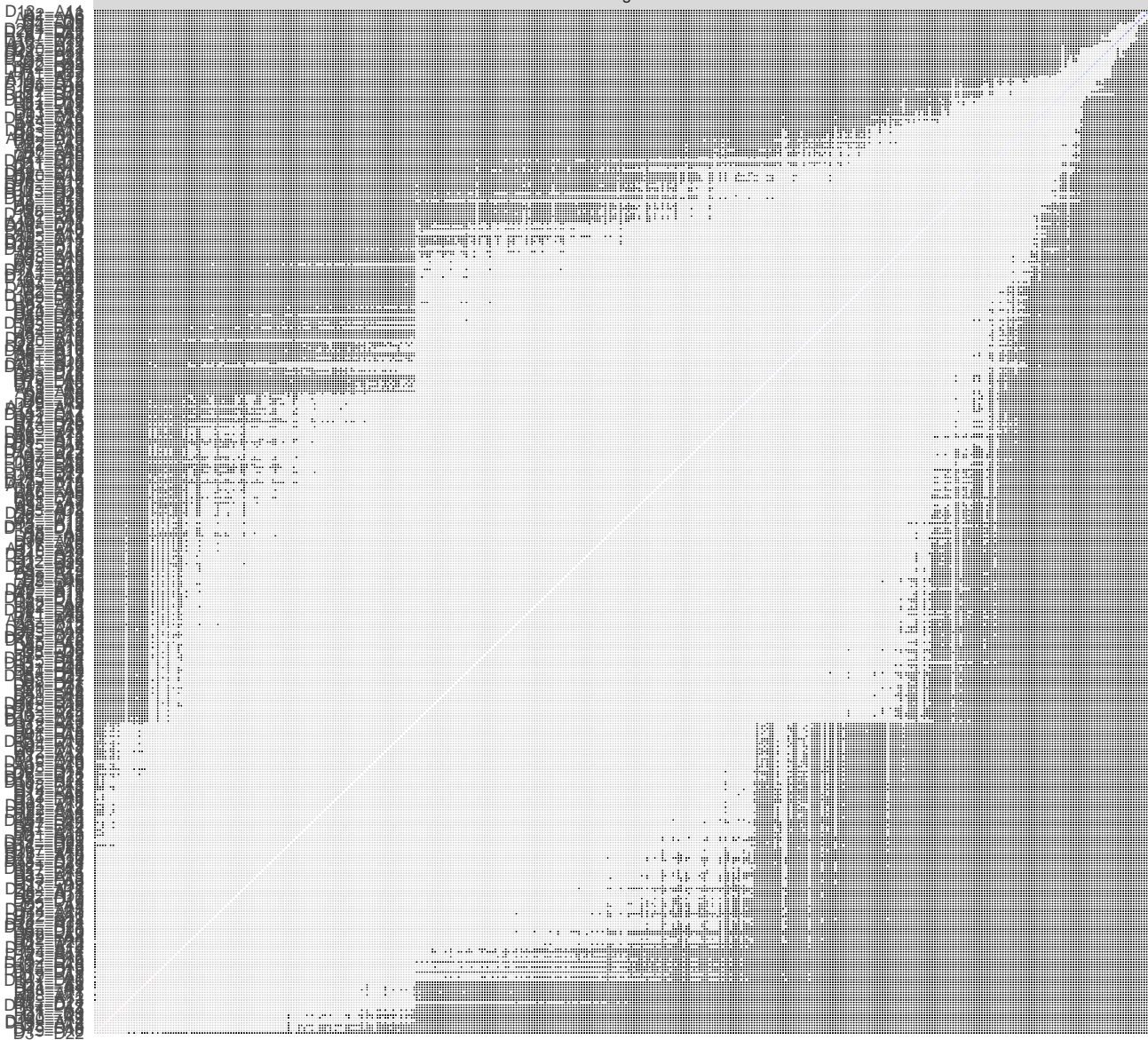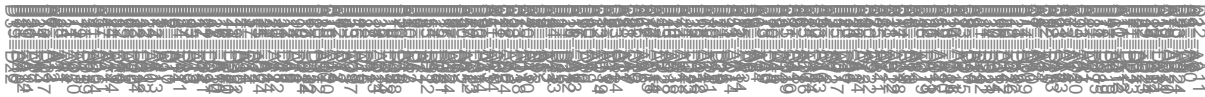

Supplement: Supplementary file 3 [file Data_Sheet_3.PDF]

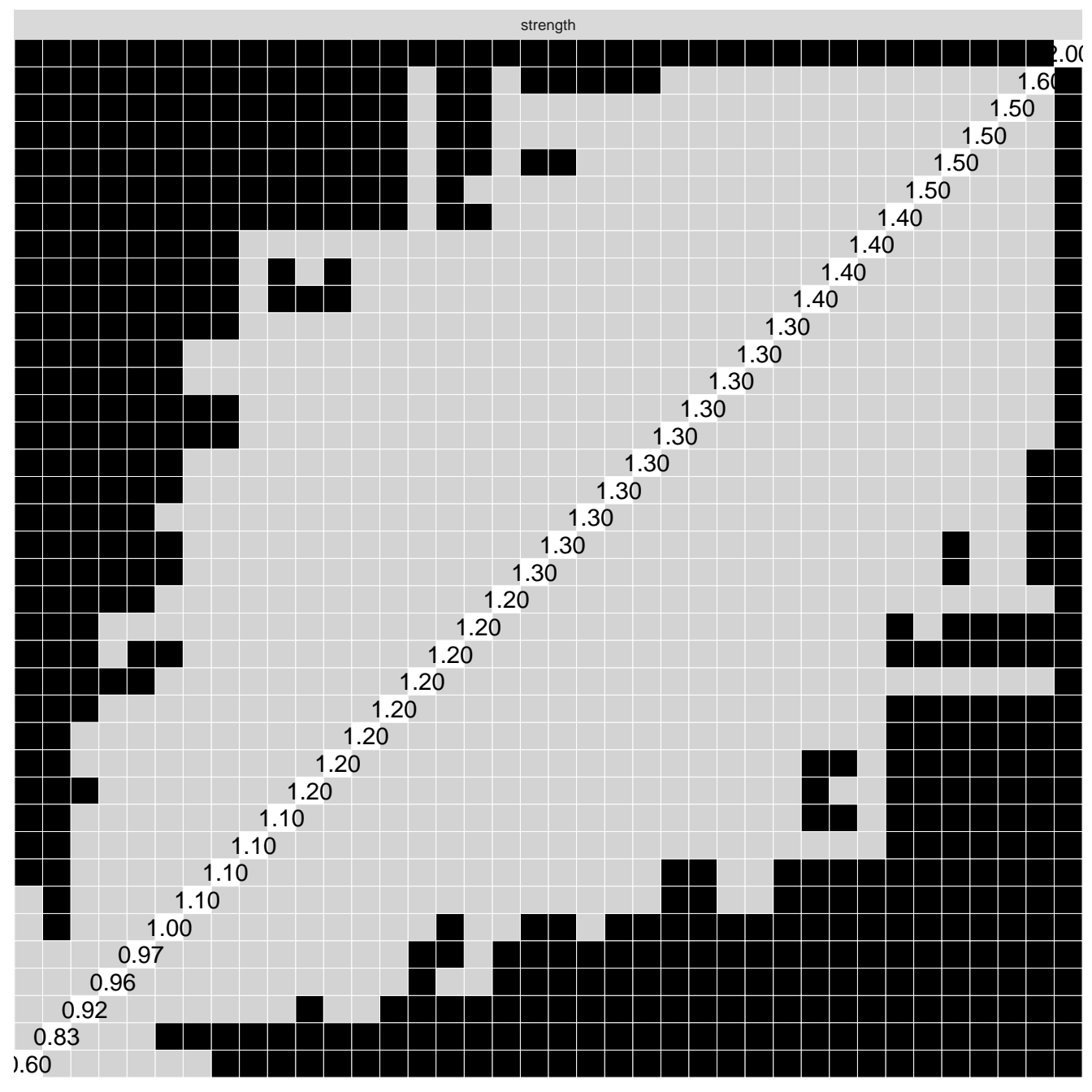

Supplement: Supplementary file 4 [file Data_Sheet_4.PDF]

● Bootstrap mean    ● Sample

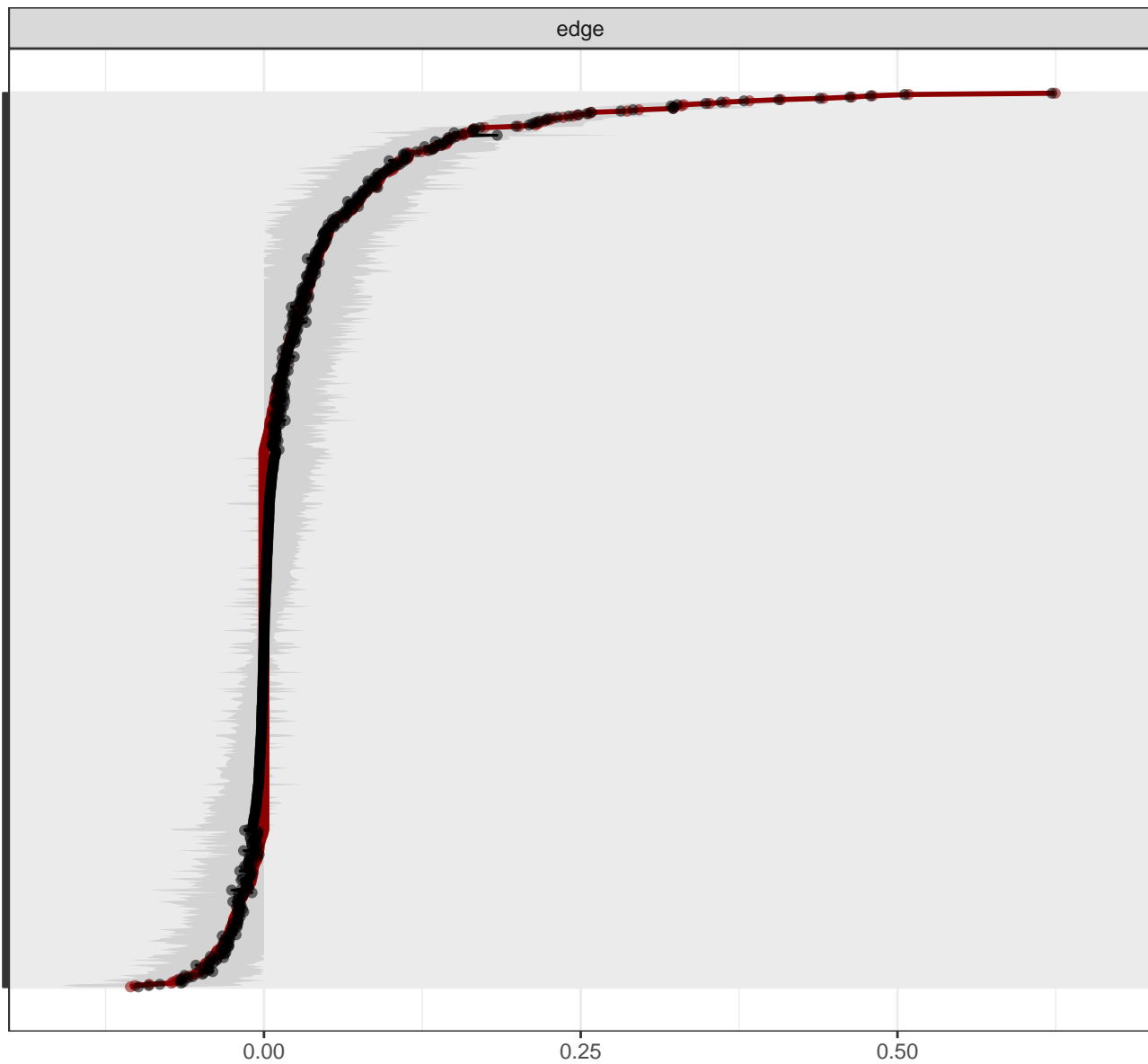

Supplement: Supplementary file 5 [file Data_Sheet_5.PDF]
